# Identification of functional enolase genes of the silkworm *Bombyx mori* from public databases with a combination of dry and wet bench processes

**DOI:** 10.1186/s12864-016-3455-y

**Published:** 2017-01-13

**Authors:** Akira Kikuchi, Takeru Nakazato, Katsuhiko Ito, Yosui Nojima, Takeshi Yokoyama, Kikuo Iwabuchi, Hidemasa Bono, Atsushi Toyoda, Asao Fujiyama, Ryoichi Sato, Hiroko Tabunoki

**Affiliations:** 1 Department of Science of Biological Production, Graduate School of Agriculture, Tokyo University of Agriculture and Technology, 3-5-8 Saiwai-cho, Fuchu, Tokyo, 183-8509 Japan; 2 Database Center for Life Science (DBCLS), Joint Support-Center for Data Science Research, Research Organization of Information and Systems (ROIS), Yata 1111, Mishima, Shizuoka 411-8540 Japan; 3 Department of Bioregulation and Biointeraction, Graduate School of Agriculture, Tokyo University of Agriculture and Technology, 3-5-8 Saiwai-cho, Fuchu, Tokyo, 183-8509 Japan; 4 Center for Information Biology, National Institute of Genetics, Yata 1111, Mishima, Shizuoka 411-8540 Japan; 5 Graduate School of Bio-Applications and Systems Engineering (BASE), 2-24-16, Naka-cho, Koganei, Tokyo, 184-8588 Japan

**Keywords:** Enolase, Reproduction, Growth regulator, *Bombyx mori*, Analysis pipeline

## Abstract

**Background:**

Various insect species have been added to genomic databases over the years. Thus, researchers can easily obtain online genomic information on invertebrates and insects. However, many incorrectly annotated genes are included in these databases, which can prevent the correct interpretation of subsequent functional analyses. To address this problem, we used a combination of dry and wet bench processes to select functional genes from public databases.

**Results:**

Enolase is an important glycolytic enzyme in all organisms. We used a combination of dry and wet bench processes to identify functional enolases in the silkworm *Bombyx mori* (BmEno). First, we detected five annotated enolases from public databases using a Hidden Markov Model (HMM) search, and then through cDNA cloning, Northern blotting, and RNA-seq analysis, we revealed three functional enolases in *B. mori*: BmEno1, BmEno2, and BmEnoC. BmEno1 contained a conserved key amino acid residue for metal binding and substrate binding in other species. However, BmEno2 and BmEnoC showed a change in this key amino acid. Phylogenetic analysis showed that BmEno2 and BmEnoC were distinct from BmEno1 and other enolases, and were distributed only in lepidopteran clusters. BmEno1 was expressed in all of the tissues used in our study. In contrast, BmEno2 was mainly expressed in the testis with some expression in the ovary and suboesophageal ganglion. BmEnoC was weakly expressed in the testis. Quantitative RT-PCR showed that the mRNA expression of BmEno2 and BmEnoC correlated with testis development; thus, BmEno2 and BmEnoC may be related to lepidopteran-specific spermiogenesis.

**Conclusions:**

We identified and characterized three functional enolases from public databases with a combination of dry and wet bench processes in the silkworm *B. mori*. In addition, we determined that BmEno2 and BmEnoC had species-specific functions. Our strategy could be helpful for the detection of minor genes and functional genes in non-model organisms from public databases.

**Electronic supplementary material:**

The online version of this article (doi:10.1186/s12864-016-3455-y) contains supplementary material, which is available to authorized users.

## Background

There are more than one million species of insects in the world. Insects can adapt to any number of environmental conditions because of their small size. Studies of insects have contributed a wealth of scientific discoveries. The i5k project, which began in 2011 [1], aims to sequence the genomes of 5000 arthropod species. This project provides genomic information for minor insect species, such as those not used experimentally (https://www.hgsc.bcm.edu/i5k-pilot-project-summary).

Researchers can now access public databases containing the genomic information of many insects for comparative analyses [2]. For our present study, we easily obtained gene sequences and analyses from large datasets, including RNA-seq results, from public databases. However, many incorrectly annotated genes are included in these databases, which can prevent the correct interpretation of gene annotations in non-model organisms. Thus, we need to develop an analysis procedure for how to select functional genes from public databases.

Enolase is a key glycolytic enzyme (2-phospho-d-glycerate hydrolase; EC 4.2.1.11). Glycolysis is responsible for the majority of energy production in all organisms. In a human study, three enolase isoenzymes were identified as homodimers composed of two alpha (also known as ENO1; Online Mendelian Inheritance in Man (OMIM), 172430), two gamma (ENO2; OMIM, 131360), or two beta (ENO3; OMIM, 131370) subunits. Isoenzyme alpha is present in most tissues, whereas the beta form is localized to the muscle and the gamma form is found only in nervous tissue [3]. A sperm-specific enolase was also identified in *Mus musculus* [4]. The ENO1 and ENO 3 sequences are well conserved in vertebrates, whereas the insect Enolase 1-like sequence is well conserved across arthropods.

In recent years, many insect enolases have been discovered. Insect enolases differ from mammalian enolases in that they have relatively low conservation among insects and show species-specific functions. For example, the enolase of the parasitic wasp, *Aphidius ervi* is expressed on the egg surface and contributes to the digestion of host proteins by promoting plasmin generation as a plasminogen receptor [5]. The expression of an enolase protein was up-regulated in the midgut of *Aedes aegypti* infected with chikungunya or dengue viruses [6]. These reports suggest that insect enolases can have many different species-specific roles. To analyze the function of an enolase, the gene sequence of the organism of interest is required.

The silkworm *Bombyx mori* is a lepidopteran insect that has been used as a model insect in agricultural research for several reasons: 1) the majority of agricultural pests are lepidopterans, 2) its genome sequence is almost completely characterized, 3) various spontaneous genetic mutants are available, and 4) the silkworm is amenable to transgenic, knock-out, and microarray technologies [7–12]. However, there have been very few reports about enolase in lepidopteran insects.

In this study, we used a combination of dry and wet bench processes to identify functional enolase genes in *B. mori* using public databases. We found two genes and one isoform of *B. mori* functional enolases and characterized their functions.

## Results

### Five enolase candidates were identified from B. mori datasets

**Fig. 1:**
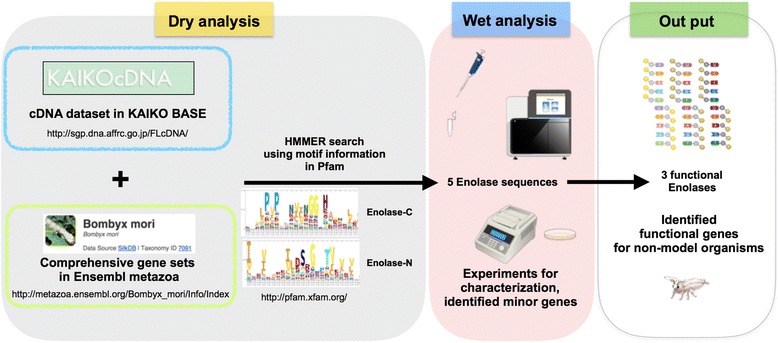
Combining dry and wet bench processes to identify functional enolases in the silkworm *B. mori*. To identify enolase sequences in *B. mori*, we performed a HMM search of public databases. We found five enolase sequences, which we then characterized using RNA-seq analysis, cDNA cloning, and RT-PCR. Finally, we determined that three enolase genes in *B. mori* were functional. The insect experimental tools and machines drawings (http://togotv.dbcls.jp/ja/pics.html ) are licensed at (http://creativecommons.org/licenses/by/4.0/deed.en)

**Fig. 2:**
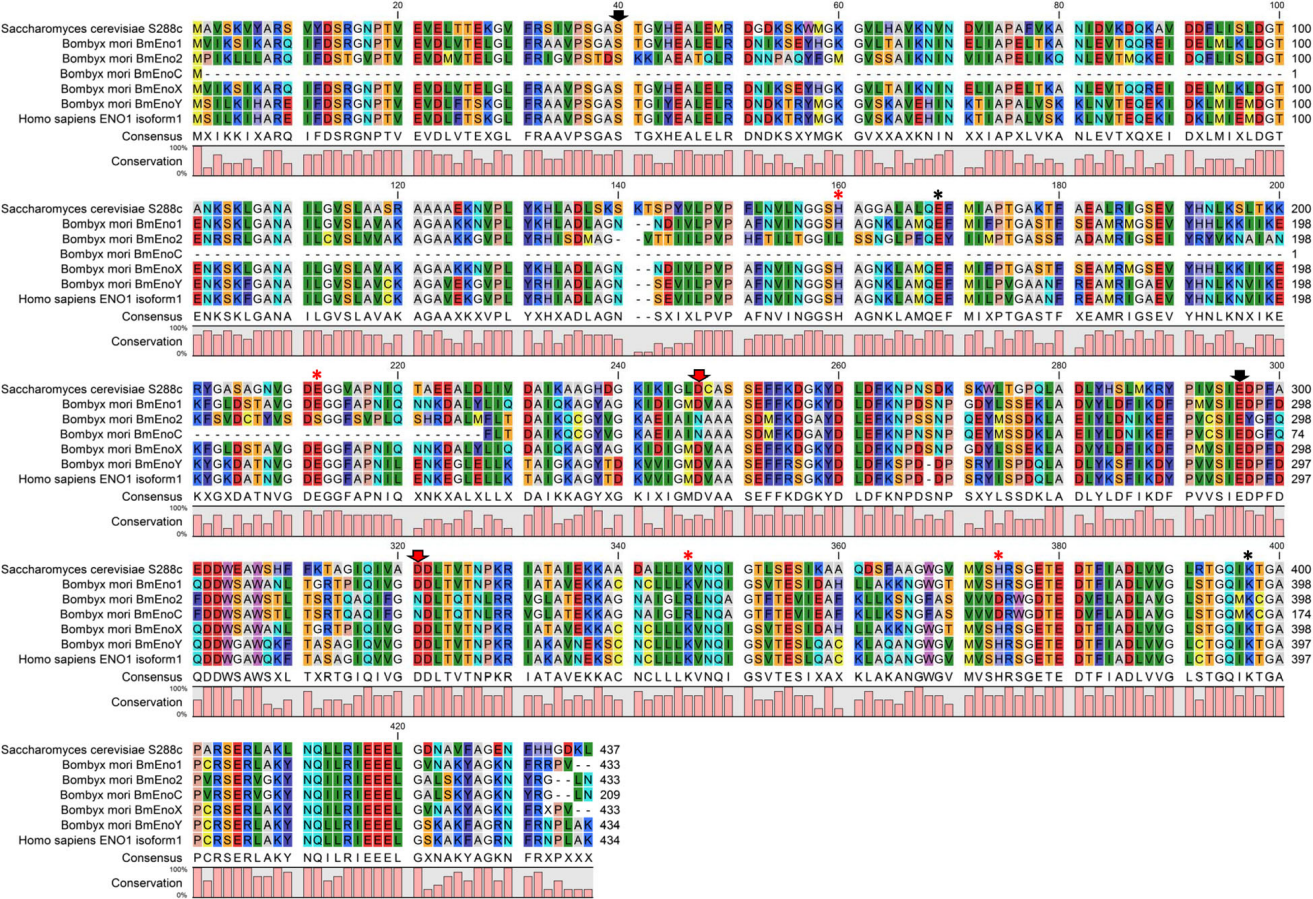
Amino acid sequence alignment of enolases from *S. cerevisiae* and *B. mori* and the alpha enolase of *H. sapiens*. Active site residues are marked with *asterisks* and metal-binding residues are indicated with *arrows. Red asterisks* and *arrows* indicate amino acid residues that differ among BmEnos. The levels of amino acid residue conservation among the various enolase sequences are graphically shown below the sequences. Residues in the alignment are colored according to the Rasmol color scheme (http://life.nthu.edu.tw/~fmhsu/rasframe/COLORS.HTM#aminocolors)

First, we searched for enolase candidate sequences in a translated database of *B. mori* Ensembl genes (14,623) and KAIKObase cDNAs (16,823) using the HMM search pipeline with two HMM profiles (enolase N-terminal domain (Enolase_N, pfam; PF03952) and enolase C-terminal domain (Enolase_C, pfam; PF00113)) retrieved from the Pfam protein family database (Fig. 1). Five sequences were revealed to encode enolases within the *B. mori* genome (Fig. 2). These sequences were annotated as enolases and were located at the following positions: BmEno1 and BmEnoX were on chr8, 11726013–11734127 (+), BmEno2 and BmEnoC were on chr26, 9181393–9182186 (+), and BmEnoY was not mapped on the *B. mori* chromosomes. The BmEno2 sequence was found in only the *B. mori* Ensembl gene dataset, and the BmEnoC sequence was found only in the KAIKObase cDNA dataset. The deduced open reading frame (ORF) of BmEno1 and BmEnoX was 1299 nucleotides long, encoding a protein with 433 amino acids, a molecular weight of 47.1 kDa, and a putative isoelectric point (pI) of 5.68. The deduced ORF of BmEno2 was 1299 nucleotides long, and encoded a protein with 433 amino acids, a molecular weight of 47.3 kDa, and a putative pI of 5.54. The deduced ORF of BmEnoC was 627 nucleotides long, encoding a protein with 209 amino acids, a molecular weight of 23.1 kDa, and a putative pI of 4.79. BmEnoC was similar to the C-terminus of the BmEno2 sequence. The deduced ORF of BmEnoY was 1302 nucleotides long, and encoded a protein with 434 amino acids, a molecular weight of 61.3 kDa, and a putative pI of 7.82.

All of these BmEno genes, except BmEnoC, contained both an enolase N-terminal domain (Enolase_N, pfam; PF03952) and enolase C-terminal domain (Enolase_C, pfam; PF00113) (Additional file 1).

BmEno1 and BmEnoX showed high homology to *Manduca sexta*, *Anopheles gambiae*, *Drosophila melanogaster*, *Apis mellifera*, *Tribolium castaneum* and *Homo sapiens* enolases. Additionally, BmEno2 and BmEnoC showed high homology to *M. sexta* enolases. The BmEnoY sequence was identical to the *H. sapiens* ENO1 sequence (Table 1).

**Table 1:** BmEno amino acid sequence homology with enolases from other species

Species	Gene ID	Identity (%)				
		BmEno1	BmEno2	BmEnoC	BmEnoX	BmEnoY
*Manduca sexta*	Msex2.06012-RA	58.4	84.3	83.7	58.4	50.2
	Msex2.06643-RA	93.1	61.2	60.3	93.1	73.4
*Anopheles gambiae*	AGAP007827-PA	82.4	55.4	53.6	82.4	76.2
*Drosophila melanogaster*	FBpp0077571	81.1	55.4	54.1	81.1	72.9
*Apis mellifera*	GB46285-PA	54.6	48.8	48.6	54.6	52.2
	GB54753-PA	74.4	56.1	52.9	74.4	67.9
*Tribolium castaneum*	TC011729-PA	77.8	53.8	50.2	77.8	69.4
	TC011730-PA	80.8	54.4	55.0	80.8	70.8
	TC012754-PA	83.4	57.7	56.5	83.4	72.2
*Homo sapiens*	NP_001419.1 (ENO1)	73.4	50.7	49.0	73.4	100.0
	NP_001966.1 (ENO2)	71.5	49.3	49.0	71.5	83.4
	NP_001967.3 (ENO3)	71.5	49.5	48.6	71.5	83.4

Therefore, we identified five putative enolase sequences from the *B. mori* gene datasets from Ensembl Metazoa and KAIKObase.

### *Analysis of* B. mori *enolase sequences*

Alignment with enolase homologs from other species showed that the deduced BmEno amino acid sequences, except for BmEno2 and BmEnoC, contained all of the conserved Ser, Glu, and Asp residues (Fig. 2, arrows). These amino acid residues are involved in the coordination of the metal-binding domain. The BmEno2 and BmEnoC sequences contained a conserved Asn residue that replaced the Asp residue as the metal-binding amino acid residue (Fig. 2, red arrows). In Fig. 2, amino acid residues (Glu, Lys, and His) related to enolase active sites, also known as substrate binding pockets, are shown with asterisks. These amino acid residues were changed to Ser, Arg, and Asp in BmEno2 and BmEnoC (Fig. 2, asterisks).

The BmEnoX amino acid sequence corresponded well with the BmEno1 amino acid sequence except for an unknown amino acid at residue 431 (shown as “X;” Fig. 2, bottom of alignment). Furthermore, the BmEnoC amino acid sequence from position 1 to 209 corresponded well with the BmEno2 amino acid sequence at positions 225 to 433 (98.6% similarity). However, the N-terminus of BmEnoC was slightly different from that of BmEno2. The entire amino acid sequence of BmEnoY corresponded with that of *H. sapiens* ENO1 (NP_001419.1) (Fig. 2).

**Fig. 3:**
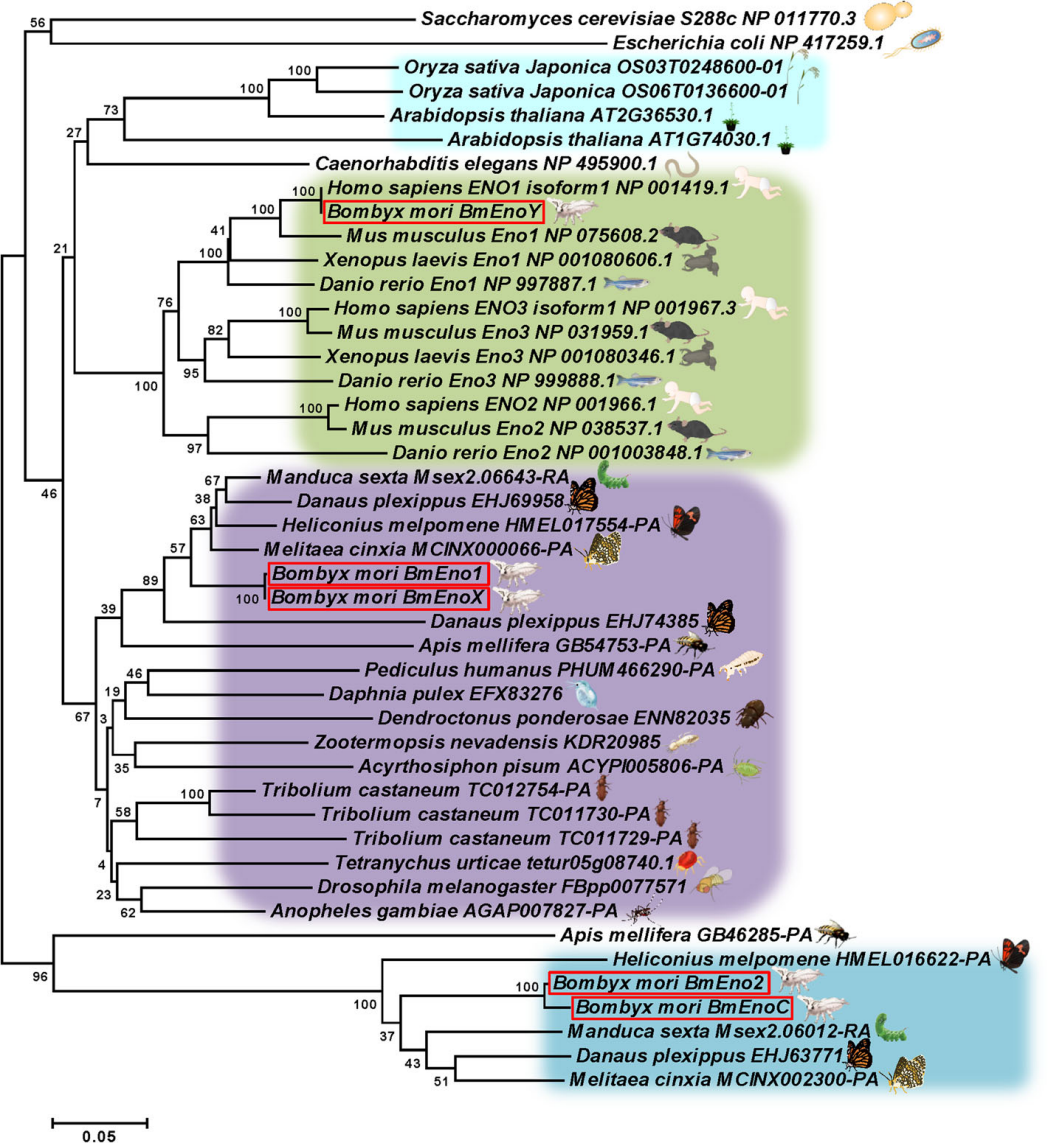
A phylogenetic tree of *B. mori* enolases and enolase proteins of other species. The amino acid sequences of the BmEnos were aligned with enolases of the species shown in Table 2. BmEnos are framed in *red*

Three enolases have been identified in vertebrates, including mammals. In the phylogenetic tree that contains amino acid sequences of the BmEnos and enlases of the species shown in Table 2, the five identified BmEnos were distributed into three clusters (Fig. 3). BmEno1 and BmEnoX were located in the same cluster close to each other. BmEnoY was distributed in the cluster that contained *H. sapiens* enolase (enolase 1 or alpha enolase). Interestingly, BmEno2 and BmEnoC were distributed in an independent cluster that contained only lepidopteran insect sequences (Fig. 3).

### cDNA cloning of BmEnos from B. mori larvae and verification with RNA-seq analysis

**Fig. 4:**
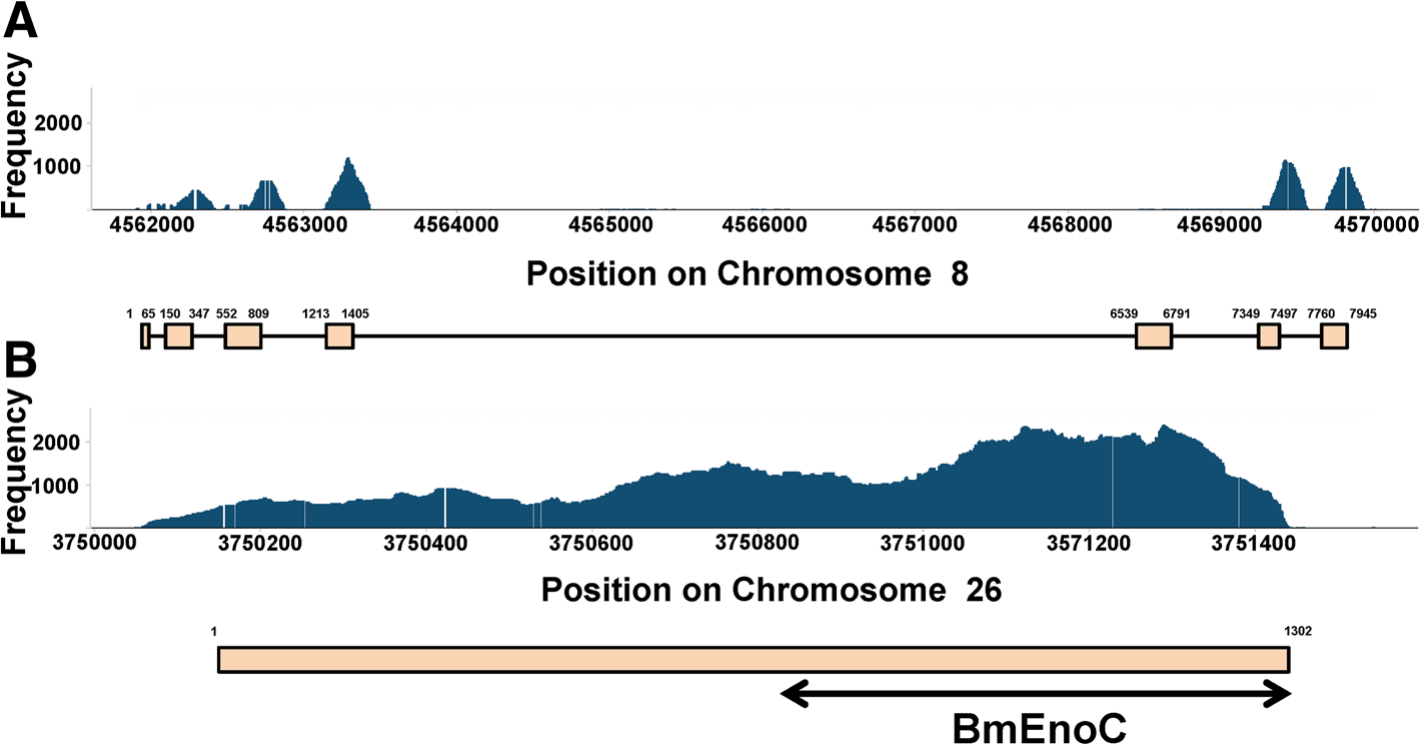
Verification of enolase mRNA expression by RNA-seq analysis. Histograms show the frequency of BmEno1 (**a**) and BmEno2 (**b**) gene expression. The *boxes* below indicate exon positions. The *black arrows* in **b** show regions similar to those of the BmEnoC sequence

Next, we cloned the BmEno cDNAs from *B. mori* larvae, and verified these sequences in the testis using RNA-seq analysis. From the cDNA cloning, we identified three BmEnos in the *B. mori* Kinshu × Showa strain: BmEno1, BmEno2, and BmEnoC. We verified the expression of the BmEno1, BmEno2, and BmEnoC mRNAs using RNA-seq analysis at single-nucleotide resolution (Fig. 4). The expression levels of BmEno2 and BmEnoC were similar, and the expression of the C-terminus was significantly increased in BmEno2 compared with the N-terminus (Fig. 4b). This comparison would not have been available without RNA-seq analysis. The nucleotide sequences of BmEno1, BmEno2, and BmEnoC were submitted to DDBJ/ENA (Accession Nos. LC170036, LC170037, and LC170038, respectively).

### Developmental stage- and tissue-specific expression patterns of BmEno mRNAs as determined by RT-PCR

**Fig. 5:**
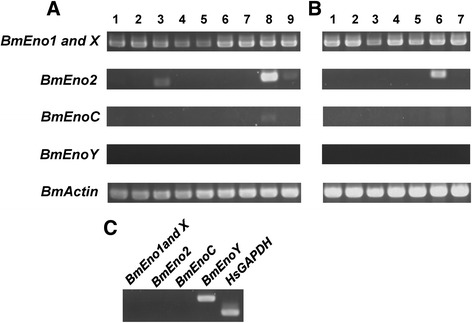
Tissue and developmental stage distribution of BmEno expression. **a** Tissue-specific expression of the BmEnos. Lane 1, brain; 2, nerve ganglion; 3, suboesophageal ganglion; 4, silk gland; 5, midgut; 6, Malpighian tubule; 7, fat body; 8, testis; and 9, ovary. Each sample was derived from a day 3 fifth-instar larva. **b** Developmental stage-specific expression of the BmEnos. Lanes 1–5, whole body, day 0 first to fifth instar larvae; 6, whole body, day 0 pupa; 7, whole body, day 0 adult. **c** Expression of the BmEnos and HsGAPDH in cDNA from HepG2 cells

The distribution of the BmEno mRNAs in different tissues during different developmental stages is shown in Fig. 5. The BmEno1 mRNA was expressed in all tissues beginning on day 3 of the fifth instar period and continued through all developmental stages. The BmEno2 mRNA was mainly localized to the testis, but also showed weak expression in the ovary and suboesophageal ganglion. The BmEno2 mRNA was detected in the whole bodies of day 0 pupae. The BmEnoC mRNA was detected only in the testis. BmEnoY was not detected in any tissue at any developmental stage (Fig. 5a and b). However, the BmEnoY mRNA was detected in the human cell line HepG2 derived cDNA library (Fig. 5c).

**Fig. 6:**
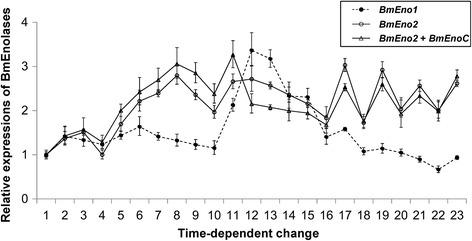
Quantitative RT-PCR confirmation of BmEno1 and BmEno2 mRNA expression in the testis. BmEno1 is marked with *black circles*, BmEno2 is marked with *white circles*, and the combination of BmEno2 and BmEnoC is marked with *white triangles*. The mRNA expression of each enolase is shown as RQ values in days 0 to 10 of the fifth-instar larval stage (Lanes 1 to 11), days 0 to 10 of the pupal stage (Lanes 12 to 22), and day 0 of the adult stage (Lane 23) (*n* = 3). The RQ value on day 0 of the fifth instar larva was set to 1 (control). *Error bars* indicate the relative minimum/maximum expression levels against mean RQ expression levels. Technical replications were performed in triplicate

We investigated the BmEno1 and BmEno2 mRNA distribution in the testis from day 0 of the fifth instar larval stage to the adult stage by quantitative RT-PCR (qRT-PCR; Fig. 6). The BmEno1, BmEno2, and BmEnoC mRNAs showed different expression patterns in the testis from day 0 fifth instar larvae to adults.

### Confirmation of BmEno isoforms

**Fig. 7:**
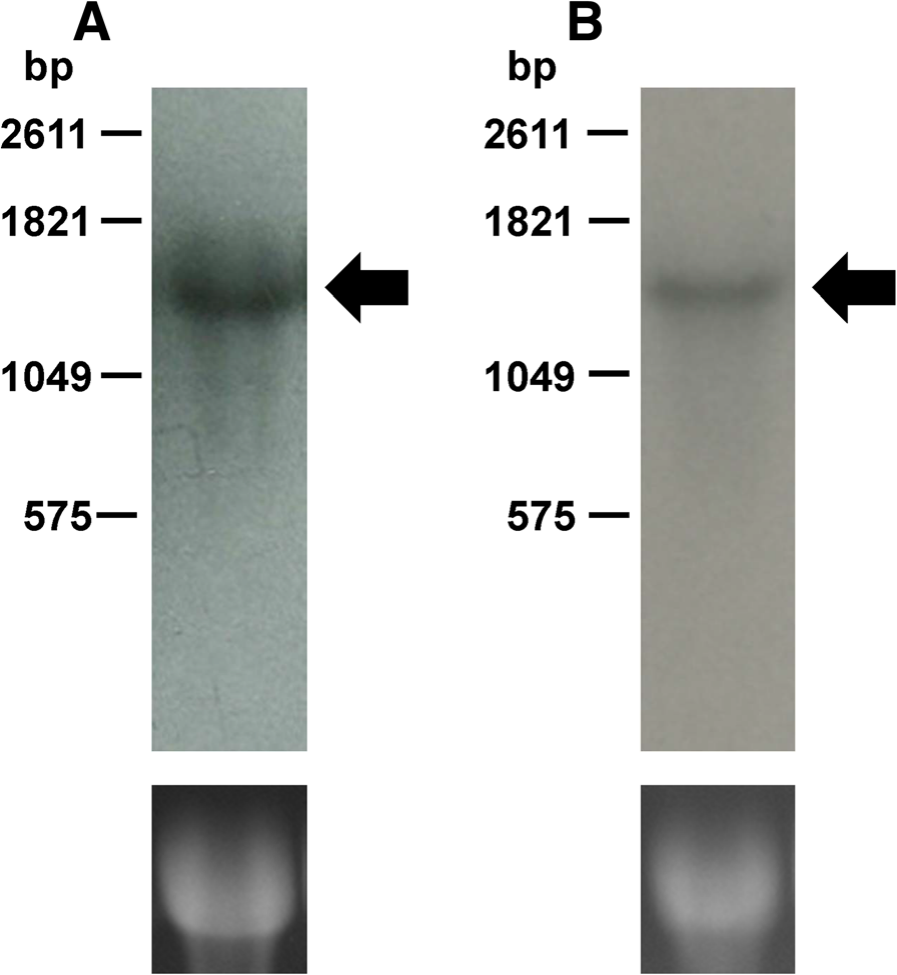
Northern blot analysis of BmEno1, BmEno2, and BmEnoC. Total RNA (12 μg per lane) isolated from the testis of day 3 fifth-instar *B. mori* larvae was analyzed by Northern blot analysis using probes that labeled BmEno1 and both BmEno2 and BmEnoC. Bands of approximately 1470 bases were identified as the BmEno1 (**a**) and BmEno2 (**b**) transcripts. A BmEnoC band was not detected. Total RNA was loaded for BmEno1, BmEno2, BmEnoC, and 18S rRNA (RNA loading control)

To verify the BmEno isoforms, a Northern blot analysis was conducted using specific probes. These probes were labeled with DIG from position 171 to 410 in the BmEno1 nucleotide sequence and from position 788 to 1022 in the BmEno2 and BmEnoC nucleotide sequences. The BmEno2 probe showed a 97.9% match with positions 116 to 350 of the BmEnoC sequence. The transcription products were detected as single bands with characteristic sizes: 1470 bases for BmEno1 (Fig. 7a) and 1470 bases for BmEno2 and BmEnoC (Fig. 7b). BmEnoC may be an isoform of BmEno2; however, we could not detect a variation in size between BmEno2 and BmEnoC with Northern blotting (Fig. 7b).

## Discussion

In this study, we obtained candidate BmEno sequences from public databases. Using a combination of dry and wet processes, we identified functional enolases in *B. mori*. These enolase amino acid sequences were composed of two domains. The N-terminus consisted of a shorter Enolase_N motif, and the C-terminus consisted of a longer Enolase_C motif. BmEnoC did not contain the Enolase_N motif.

The His 159, Glu 168, Glu 211, Lys 345, His 373, and Lys 396 amino acid residues are required for *S. cerevisiae* enolase activity. Amino acid residues Ser 39, Asp 246, Glu 295, and Asp 320 were identified as critical for metal-binding in *S. cerevisiae* enolase [13–16]. BmEno1 also contained these active residues (Fig. 2, asterisks) and metal-binding residues (Fig. 2, arrows). However, BmEno2 and BmEnoC had different amino residues from BmEno1 at the metal-binding and substrate-binding sites (Fig. 2, red asterisks and arrows). The Chinese oak moth (*Antheraea pernyi*) is a lepidopteran insect, and its enolaseI and enolaseII genes corresponded well with BmEno1 and BmEno2. *A. pernyi* enolaseII contains the same metal-binding and substrate-binding amino acid residues as BmEno2 and BmEnoC [17]. Our phylogenetic analysis showed that BmEno1 was close to the cluster containing insect enolases, such as those from *D. melanogaster*, *T. castaneum* and *A. mellifera*. BmEno2 and BmEnoC were located in a lepidopteran-specific cluster. Sequences that belong to this cluster have residues that differ from the conserved residues necessary for enolase activity (Additional file 2). Thus, lepidopteran-specific enolases may have glycolytic enzyme activity that does not require the aforementioned conserved enolase residues.

cDNA cloning showed that the BmEno1 amino acid sequence corresponded with that of BmEnoX. We also confirmed that the chromosome positions of BmEno1 and BmEnoX were identical. As a result, only one transcript was detected as BmEno1. These results suggest that the BmEnoX sequence was misread and incorrectly registered in the public database.

The chromosome positions of BmEno2 and BmEnoC were also nearly identical. However, the BmEnoC sequence differed from that of BmEno2 by three amino acid residues. cDNA cloning and RNA-seq analysis showed BmEnoC is likely an isoform of BmEno2. RT-PCR analysis showed that the BmEno2 mRNA was mainly expressed in the testis, followed by the suboesophageal ganglion, and also a little bit in the ovary. BmEnoC was only expressed in the testis. To examine the physiological functions of BmEno2 and BmEnoC in the testis, we performed qRT-PCR on testis from day 0 fifth-instar larvae to adults. BmEno2 and BmEnoC were highly expressed from day 5 of the fifth-instar larval stage to the prepupal stage. When spermiogenesis occurs from the end of larval developmental stage to the prepupal developmental stage, the testis becomes hypertrophied in *B. mori* [18]. The ecdysone titer is increased in *B. mori* at this time [19]. Thus, the expression of BmEno2 and BmEnoC may correlate with spermiogenesis in *B. mori*.

Almost all lepidopteran insects have two kinds of sperm: apyrene and eupyrene sperm [20, 21]. Both sperm types are essential for fertilization in lepidopteran insects, but the role of this evolutionarily-conserved system in fertilization remains unclear. Furthermore, a sperm-specific enolase was reported to control sperm formation and mobility in *M. musculus* [22].

In this study, we found differences in the mRNA expression of BmEno2 and BmEnoC. The BmEno2 mRNA was expressed in the ovary and suboesophageal ganglion. Pheromone biosynthesis activating neuropeptide (PBAN) is secreted from the suboesophageal ganglion in *B. mori*, and affects the pheromone glands of female moths and stimulates the biosynthesis of a sex pheromone [23–25]. Diapause hormone (DH) is also secreted from the suboesophageal ganglion and promotes embryonic diapause [26–28]. DH also stimulates the prothoracic gland and promotes ecdysteroid generation, which controls molting and metamorphosis [29]. Future studies should examine the function of BmEno2 outside of reproduction. Based on these data, BmEnoC might be an isoform of BmEno2 that has a different function.

BmEnoY mRNA expression was not detected by RT-PCR in any *B. mori* tissue at any developmental stage. However, the BmEnoY mRNA was expressed in the human cell line HepG2. Sequence analysis of BmEnoY showed that its amino acid sequence completely matched that of *H. sapiens* alpha enolase. Thus, BmEnoY might be a result of contamination by human alpha enolase that was incorrectly registered in the public database. In conclusion, our results suggest BmEno1, BmEno2, and BmEnoC are functional enolases in *B. mori*.

In this study, we performed a pipeline analysis using a combination of dry and wet bench processes. Using a molecular biological approach, we identified functional enolases in *B. mori*. BmEno1 was conserved across species. However, BmEno2 and BmEnoC appear to have a lepidopteran-specific function rather than a glycolytic enzyme function. Notably, enolase functions as a dimer. Therefore, research on how the enolases characterized in this study combine and function is needed. Future studies should compare the expression and enzymatic activities of the dimer proteins in different tissues and developmental stages. The key BmEno2 amino acid residues partially differed from those in BmEno1, and may play an important role in enzyme activity and metal binding.

## Conclusions

We identified three *B. mori* enolases using a combination of dry and wet bench processes. These BmEnos have different functions within the tissues of *B. mori*. At some point, incomplete transcripts or uncorrected data were registered in public databases. If we can resolve these database errors using wet bench processes, then the usefulness of public databases will increase for all users. All public databases provide a wealth of information for future scientific research. Thus, we proposed a procedure for how to identify active genes from public databases in this study. It is important that public databases are regularly maintained by users. Our combination of dry and wet experiments is useful for the detection of minor genes and declared functional genes of non-model organisms in public databases.

## Methods

### Insects

The *B. mori* hybrid strain Kinshu × Showa used in this study was supplied by Ueda-Sha Co. Ltd. (Nagano, Japan). Silkworm larvae were reared on the artificial diet silk-mate 2S (Nosan, Tsukuba, Japan). Insects were maintained at 25 °C with a 12-h light/dark cycle. The *B. mori* strain o751 (wild-type) used in the RNA-seq analysis was obtained from the Institute of Genetic Resources, Faculty of Agriculture, Kyushu University (NBRP silkworm database: http://silkworm.nbrp.jp/index_en.html).

### *Identification of* B. mori *enolase sequences by HMM search and bioinformatics*

The HMM search program in the HMMER package (version 3.1b1) [30] was used to detect enolase candidates. HMM profiles of the enolase N-terminal domain (Enolase_N, PF03952) and C-terminal domain (Enolase_C, PF00113) in the Pfam 27.0 database [31] were queried against deduced protein sequences in a *B. mori* Ensembl Gene dataset [32] and a cDNA dataset [9] with default parameters.

A search for enolase orthologs among the genes of the following species was conducted using BLAST methods: *D. melanogaster*, *M. sexta*, *A. gambiae*, *A. mellifera*, *T. castaneum,* and *H. sapiens*. Global homology searches were conducted using Genetyx ver. 10 (Genetyx Co. Ltd., Tokyo, Japan). A phylogenic analysis was conducted using MEGA ver. 7 [33]. A protein motif search was conducted using SMART (http://smart.embl-heidelberg.de/). The alignment of the BmEno amino acid sequences and enolase orthologs from other species was conducted using CLC Sequence viewer 7.6.1 (CLC Bio Japan Inc. Tokyo, Japan). All analyses were performed with default parameters for the software.

### Purification of total RNA and cDNA synthesis from different tissues and whole-body samples

Various tissues were dissected from day 3 fifth-instar larvae: brain, nerve ganglion, midgut, silk gland, fat body, Malpighian tubules, testis, and ovary. These tissues were stored at −80 °C until use. Larval, pupal, and adult whole bodies were also used for total RNA purification. Whole bodies were freeze-dried using a freeze drier (TAITECH Co. Ltd., Tokyo, Japan) for 12 h. Tissues and freeze-dried whole bodies were weighed and homogenized with lysis buffer from a PureLink® RNA extraction kit (Thermo Fisher Scientific Inc., Valencia, CA, USA) and then centrifuged at 13,000 × g for 10 min. Next, the supernatants were collected and processed for RNA purification according to the manufacturer’s instructions. Purified total RNA (1 μg) was processed for cDNA synthesis using a PrimeScript™ 1st strand cDNA Synthesis Kit (Takara Co. Ltd., Tokyo, Japan).

### cDNA cloning of B. mori enolases

BmEno cDNA sequences were amplified by PCR using KOD–plus-neo polymerase (Toyobo Co. Ltd., Tokyo, Japan) with specific primers (Table 3). The amplified products were cloned into the cloning vector Topo-p2T (Invitrogen, Van Allen Way, Carlsbad, CA, USA) and then transformed into *Escherichia coli* XL-1 Blue (Toyobo). The purified vectors were processed for sequencing by the dideoxynucleotide chain termination method on an ABI PRIZM 3100 Genetic Analyzer (Applied Biosystems, Tokyo, Japan).

**Table 2:** Enolase homologs in species used for the phylogenic tree

Species	Gene ID
*Homo sapiens*	NP_001419.1
	NP_001966.1
	NP_001967.3
*Mus musculus*	NP_031959.1
	NP_038537.1
	NP_075608.2
*Xenopus laevis*	NP_001080606.1
	NP_001080346.1
*Danio rerio*	NP_997887.1
	NP_001003848.1
	NP_999888.1
*Manduca sexta*	Msex2.06012-RA
	Msex2.06643-RA
*Melitaea cinxia*	MCINX000066-PA
	MCINX002300-PA
*Danaus plexippus*	EHJ63771
	EHJ69958
	EHJ74385
*Heliconius melpomene*	HMEL016622-PA
	HMEL017554-PA
*Anopheles gambiae*	AGAP007827-PA
*Drosophila melanogaster*	FBpp0077571
*Apis mellifera*	GB46285-PA
	GB54753-PA
*Daphnia pulex*	EFX83276
*Zootermopsis nevadensis*	KDR20985
*Tribolium castaneum*	TC011729-PA
	TC011730-PA
	TC012754-PA
*Tetranychus urticae*	tetur05g08740.1
*Dendroctonus ponderosae*	ENN82035
*Pediculus humanus*	PHUM466290-PA
*Acyrthosiphon pisum*	ACYPI005806-PA
*Caenorhabditis elegans*	NP_495900.1
*Escherichia coli*	NP_417259.1
*Saccharomyces cerevisiae* S288c	NP_011770.3
*Oryza sativa Japonica*	OS06T0136600-01
	OS03T0248600-01
*Arabidopsis thaliana*	AT1G74030.1
	AT2G36530.1

**Table 3:** Primers used for Northern blot analysis (A), RT-PCR (B), and quantitative RT-PCR (C)

Gene	Forward primer	Reverse primer
A		
BmEno1	5'-TCATGGCAAGGGAGTTTTGA-3'	5'-AAATCAGCCAAGTGCTTGTA-3'
BmEno2 and BmEnoC	5'-ATCCGAACTCAAATCCTCAG-3'	5'-ATCGCATTTCCCGCTTTTTT-3'
B		
BmEno1	5'-GTAATAAAATCAATCAAGGCTCG-3'	5'-TTAGACCGGTCTACGGAAGTTCT-3'
BmEno2	5'-TCCGGAGGATTTTCTGTGCC-3'	5'-GCGGAGATTAGTTTGAGTAA-3'
BmEnoY	5'-CCATGCAGGAGTTCATGATCCT-3'	5'-CTGGGTAGTCCTTGATGAAGGA-3'
BmActin	5'-TATCGCCGACAGGATGCAGAAGGA-3'	5'-TAGAAGCACTTGCGGTGAACGATG-3'
HsGAPDH	5'-GTCAGTGGTGGACCTGACCT-3'	5'-TGCTGTAGCCAAATTCGTTG3'
C		
BmEno1	5'-AAGGTCAATCAGATCGGTAGCG-3'	5'-ACTACCAGGTCGGCAATAAAGG-3'
BmEno2	5'-GGAGCTAACGCGATTCTTTGTG-3'	5'-ACGCCTGCCATATCCGATATG-3'
BmEno2 and BmEnoC	5'-AATAAATGCTGCCGCCAGTG-3'	5'-GGCAAGTTTATCCGACGACATG-3'
RP49	5'-ACGGTTCCAACAAGAAGACC-3'	5'-AAGAGACACCATGAGCGATCTC-3'

### Tissue and developmental distribution analysis by RT-PCR

The tissue distribution of the BmEno genes was determined in the brain, nerve ganglion, suboesophageal ganglion, midgut, silk gland, fat body, Malpighian tubules, testis, and ovary of day 3 fifth-instar larvae. The distribution of the BmEno genes in the whole bodies of first instar to fifth instar larvae, pupae, and adults were determined. All samples were processed for extraction of total RNA and cDNA synthesis as previously described. Reverse transcriptase (RT)-PCR was performed with specific primers (Table 3) using AmpliTaq Gold® 360 Master Mix (Thermo Fisher Scientific Inc.) according to the manufacturer’s protocol. *B. mori* actin (BmActin, Gene ID 187281813) was used as an endogenous control.

### Northern blot analysis

Total RNA derived from the testis of day 3 fifth-instar larvae was used. Total RNA (12 μg) was separated on a 1.5% agarose and 6% formaldehyde gel and stained with ethidium bromide. Next, the gel was transferred to a nylon membrane. DIG-labeled probes were synthesized using a PCR DIG probe synthesis kit (Roche Diagnostics, Mannheim, Germany) with specific primers (Table 3). After pre-hybridization, the membrane was hybridized with DIG-labeled probes at 50 °C overnight. The specific reaction was visualized on Kodak XOMAT AR X-ray film using a DIG chemiluminescence detection kit (Roche Diagnostics). 18S ribosomal RNA (rRNA) was used as a control. The mRNA size of BmEno genes was calculated using the image analysis software CS analyzer Ver. 3.0. A calibration curve was determined using the mobility of the DIG RNA ladder marker (Roche Diagnostics).

### qRT-PCR

To quantify RNA expression levels, 1 μg of total RNA from pooled testis tissue dissected from day 0 fifth-instar larvae to day 0 adults (*n* = 3 each) was used for cDNA synthesis. qRT-PCR was performed in a 20 μl reaction volumes with 0.5 μl of the cDNA template and primers (Table 3) with a KAPA SYBR Fast qRT-PCR Kit (Nippon Genetics Co., Ltd., Tokyo, Japan) in accordance with the manufacturer’s instructions. qRT-PCR was performed on a Step One plus Real-Time PCR System (Applied Biosystems, Foster City, CA, USA) following the Delta-Delta Ct method. Ribosome protein 49 (GeneID: 778453) was used as an endogenous reference for the standardization of RNA expression levels, and all data were calibrated against universal reference data. Relative quantification (RQ) values represent the relative expression level against a reference sample. All samples were assayed in triplicate as technical replications.

### RNA-seq analysis

Total RNA was isolated from the testis of day 3 fifth-instar larvae of the *B. mori* o751 wild type strain using a PureLink® RNA extraction kit (Thermo Fisher Scientific Inc.) according to the manufacturer’s protocol. The quality of RNA was assessed using an Agilent Bio-analyzer 2100 (Agilent Technologies, Santa Clara, CA, USA). Paired-end sequencing cDNA libraries were constructed with 4 μg of total RNA from o751 wild type testis samples (*n* = 3) with a Truseq RNA Sample Preparation Kit Set A (Illumina Inc., San Diego, CA, USA) according to the manufacturer’s protocol. RNA-seq was performed using a HiSeq 2500 system (Illumina Inc.). The data quality of the fastq files was verified with the FastQC tool (Babraham Bioinformatics, http://www.bioinformatics.babraham.ac.uk/projects/fastqc/). The 44 M paired-end reads (2 × 150 bp) were mapped to the reference *B. mori* genomes available in the Ensembl Genome database [30, 32] using the Tophat program version 2.0.13 with default parameters [34]. BAM formatted files generated by Tophat were sorted and indexed using SAMtools [35] and then converted to Wiggle track format (WIG) files using the bam2wig software (https://github.com/MikeAxtell/bam2wig). This allowed us to visualize the density of reads mapped to the specific region of interest. Histograms of mapped reads were generated using the Spotfire Cloud software with TIBCO Spotfire’s “Better World” program license (TIBCO Software, Inc., Palo Alto, CA, USA) (http://spotfire.tibco.com/better-world-donation-program/).

## Additional file

Additional file 1: Domain structure of *B. mori* enolases. Amino acid sequences of *B. mori* enolases (BmEnos) were analyzed by SMART (http://smart.embl-heidelberg.de/). The enolase_N (blue colored) or enolase_C (red colored) domain is shown by squares. The upper number is the amino acid number.
Additional file 2: Amino acid sequence alignment of Lepidopteran enolases. Active site residues are marked with asterisks, and metal-binding residues are labeled with arrows. Red asterisks and arrows indicate amino acid residues that differ among *B. mori* enolases (BmEnos). The concervation of amino acid residues among the various enolase sequences is graphically shown below the sequence. The residues in the alignment are colored according to the Rasmol color scheme (http://life.nthu.edu.tw/~fmhsu/rasframe/COLORS.HTM#aminocolors).

## References

[1] i5K Consortium (2013). The i5K Initiative: advancing arthropod genomics for knowledge, human health, agriculture, and the environment. J Hered.

[2] Poelchau M, Childers C, Moore G, Tsavatapalli V, Evans J, Lee CY, Lin H, Lin JW, Hackett K (2015). The i5k Workspace@NAL--enabling genomic data access, visualization and curation of arthropod genomes. Nucleic Acids Res.

[3] Giallongo A, Feo S, Moore R, Croce CM, Showe LC (1986). Molecular cloning and nucleotide sequence of a full-length cDNA for human alpha enolase. Proc. Nat. Acad. Sci..

[4] Edwards YH, Grootegoed JA (1983). A sperm specific enolase. J Reprod Fertil.

[5] Nguyen TTA, Magnoli I, Cloutier C, Michaud D, Muratori F, Hance T (2013). Early presence of an enolase in the oviposition injecta of the aphid parasitoid Aphidius ervi analyzed with chitosan beads as artificial hosts. J Insect Physiol.

[6] Tchankouo-Nguetcheu S, Khun H, Pincet L, Roux P, Bahut M, Huerre M, Guette C, Choumet V. Differential protein modulation in Midguts of Aedes aegypti infected with chikungunya and dengue 2 viruses. PLoS One. 2010. doi:10.1371/journal.pone.0013149. 10.1371/journal.pone.0013149 PMC2950154 20957153

[7] Mita K, Kasahara M, Sasaki S (2004). The genome sequence of silk- worm, *Bombyx mori*. DNA Res.

[8] Xia Q, Zhou Z, Lu C (2004). A draft sequence for the genome of the domesticated silkworm (*Bombyx mori*). Science.

[9] Suetsugu Y, Futahashi R, Kanamori H (2013). Large scale full-length cDNA sequencing reveals a unique genomic landscape in a lepidopteran model insect, *Bombyx mori*. G3 (Bethesda).

[10] Tomita M, Munetsuna H, Sato T, Adachi T, Hino R, Hayashi M, Shimizu K, Nakamura N, Tamura T, Yoshizato K (2003). Transgenic silkworms produce recombinant human type III procollagen in cocoons. Nat Biotechnol.

[11] Wang F, Ma S, Xu H, Duan J, Wang Y, Ding H, Liu Y, Wang X, Zhao P, Xia Q (2013). High-efficiency system for construction and evaluation of customized TALENs for silkworm genome editing. Mol Genet Genomics.

[12] Xia Q, Cheng D, Duan J, Wang G, Cheng T, Zha X, Liu C, Zhao P, Dai F, Zhang Z, He N, Zhang L, Xiang Z (2007). Microarray-based gene expression profiles in multiple tissues of the domesticated silkworm, *Bombyx mori*. Genome Biol.

[13] Brewer JM, Holland MJ, Lebioda L (2000). The H159A mutant of yeast enolase 1 has significant activity. Biochem Biophys Res Commun.

[14] Reed GH, Poyner RR, Larsen TM, Wedekind JE, Rayment I (1996). Structural and mechanistic studies of enolase. Curr Opin Struct Biol.

[15] Brewer JM, Glover CVC, Holland MJ, Lebioda L (1997). Effect of site-directed mutagenesis of His373 of yeast enolase on some of its physical and enzymatic properties. Biochimica et Biophysica Acta (BBA)- Protein Structure and Molecular Enzymology.

[16] Larsen TM, Wedekind JE, Rayment I, Reed GH (1996). A carboxylate oxygen of the substrate bridges the magnesium ions at the active site of enolase: structure of the yeast enzyme complexed with the equilibrium mixture of 2-phosphoglycerate and phosphoenolpyruvate at 1.8 Å resolution. Biochemistry.

[17] Liu Y, Li Y, Wang H, Xia R, Li X, Wan H, Qin L, Jiang D, Lu C, Xiang Z (2010). cDNA cloning and expression pattern of two enolase genes from the Chinese oak silkworm, Antheraea pernyi. Acta Biochim Biophys Sin (Shanghai).

[18] Sado T (1963). Spermatogenesis of the silkworm and its bearing on radiation induced sterility. I. Journal of the Faculty of Agriculture, Kyushu University.

[19] Kamimura M, Takahashi M, Kikuchi K, Reza AM, Kiuchi M (2007). Tissue-specific regulation of juvenile hormone esterase gene expression by 20-hydroxyecdysone and juvenile hormone in Bombyx mori. Arch Insect Biochem Physiol.

[20] Toyama K (1894). On the spermatogenesis of the silkworm. Bull Coll Agric Tokyo Imp Univ.

[21] Meves F (1903). Über oligopyrene und apyrene Spermien und uber ihre Entstehung, nach Beobachtungen an Paldina und Pygarera. Arch Micro Anat.

[22] Nakamura N, Dai Q, Williams J, Goulding EH, Willis WD, Brown PR, Eddy EM (2013). Disruption of a spermatogenic cell-specific mouse Enolase 4 (Eno4) gene causes sperm structural defects and male infertility. Biol Reprod.

[23] Ando T, Arima R, Uchiyama M, Nagasawa H, Inoue T, Suzuki A (1988). Pheromone biosynthesis activating neuropeptide hormone in heads of the silkworm moth. Agric Biol Chem.

[24] Hull JJ, Ohnishi A, Moto K, Kawasaki Y, Kurata R, Suzuki MG, Matsumoto S (2004). Cloning and characterization of the pheromone biosynthesis activating neuropeptide receptor from the silkmoth, bombyx mori significance of the carboxyl terminus in receptor internalization. Journal of Biological Chemistry.

[25] Kawai T, Katayama Y, Guo L, Liu D, Suzuki T, Hayakawa K, Lee JM (2014). Identification of functionally important residues of the silkmoth pheromone biosynthesis-activating neuropeptide receptor, an insect ortholog of the vertebrate neuromedin U receptor. Journal of Biological Chemistry.

[26] Fukuda S (1952). Function of the pupal brain and suboesophageal ganglion in the production of non-diapause and diapause eggs in the silkworm. Annotationes zoologicae Japonenses.

[27] Homma T, Watanabe K, Tsurumaru S, Kataoka H, Imai K, Kamba M, Niimi T, Yamashita O, Yaginuma T (2006). G protein-coupled receptor for diapause hormone, an inducer of bombyx embryonic diapause. Biochem Biophys Res Commun.

[28] Shiomi K, Takasu Y, Kunii M, Tsuchiya R, Mukaida M, Kobayashi M, Sezutsu H, Ichida Takahama M, Mizoguchi A (2015). Disruption of diapause induction by TALEN-based gene mutagenesis in relation to a unique neuropeptide signaling pathway in bombyx. Sci Rep.

[29] Watanabe K, Hull JJ, Niimi T, Imai K, Matsumoto S, Yaginuma T, Kataoka H (2007). FXPRL-amide peptides induce ecdysteroidogenesis through a G-protein coupled receptor expressed in the prothoracic gland of bombyx mori. Mol Cell Endocrinol.

[30] Finn RD, Clements J, Eddy SR (2011). HMMER web server: interactive sequence similarity searching. Nucleic Acids Res.

[31] Finn RD, Bateman A, Clements J, Coggill P, Eberhardt RY, Eddy SR (2013). Pfam: the protein families database. Nucleic Acids Res.

[32] Kersey PJ, Allen JE, Armean I, Boddu S, Bolt BJ, Carvalho-Silva D, Christensen M, Davis P, Falin LJ, Grabmueller C, Humphrey J, Kerhornou A, Khobova J, Aranganathan NK, Langridge N, Lowy E, McDowall MD, Maheswari U, Nuhn M, Ong CK, Overduin B, Paulini M, Pedro H, Perry E, Spudich G, Tapanari E, Walts B, Williams G, Tello-Ruiz M, Stein J, Wei S, Ware D, Bolser DM, Howe KL, Kulesha E, Lawson D, Maslen G, Staines DM (2015). Staines. Ensembl genomes 2016: more genomes, more complexity. Nucleic Acids Res.

[33] Tamura K, Stecher G, Peterson D, Filipski A, Kumar S (2013). MEGA6: Molecular Evolutionaly Genetics Analysis version 6.0. Mol Biol Evol.

[34] Kim D, Pertea G, Trapnell C, Pimentel H, Kelley R, Salzberg SL (2013). TopHat2: accurate alignment of transcriptomes in the presence of insertions, deletions and gene fusions. Genome Biol.

[35] Li H, Handsaker B, Wysoker A, Fennell T, Ruan J, Homer N, Marth G, Abecasis G, Durbin R, 1000 Genome Project Data Processing Subgroup (2009). The sequence alignment/map (SAM) format and SAMtools. Bioinformatics.

