# Supplementary figures and images for: Identification of functional enolase genes of the silkworm Bombyx mori from public databases with a combination of dry and wet bench processes
Source: BMC Genomics. 2017 Jan 13;18:83. doi: 10.1186/s12864-016-3455-y (PMC5237310; doi:10.1186/s12864-016-3455-y)

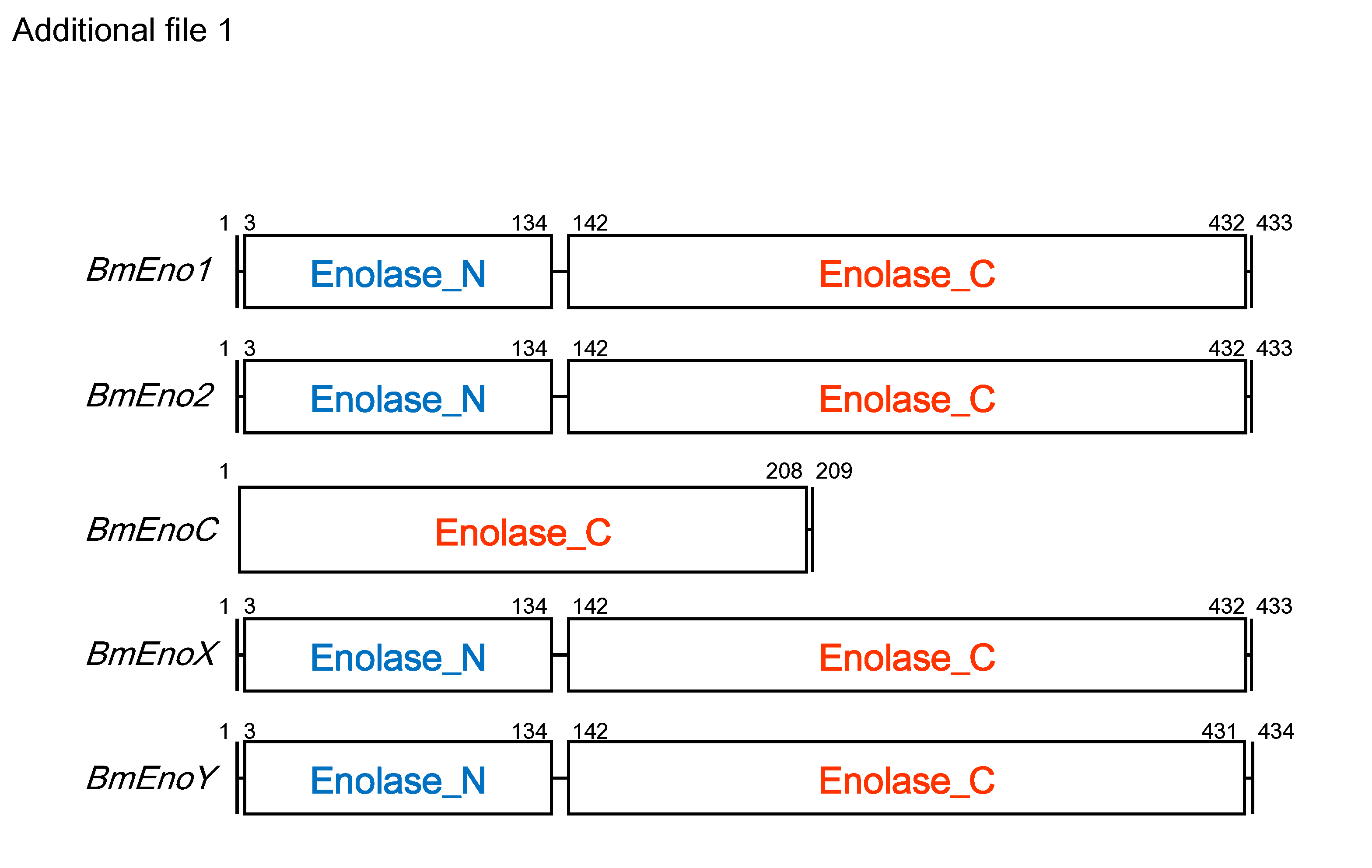

Supplement: Additional file 1: — Domain structure of B. mori enolases. Amino acid sequences of B. mori enolases (BmEnos) were analyzed by SMART (http://smart.embl-heidelberg.de/). The enolase_N (blue colored) or enolase_C (red colored) domain is shown by squares. The upper number is the amino acid number. [file 12864_2016_3455_MOESM1_ESM.docx]

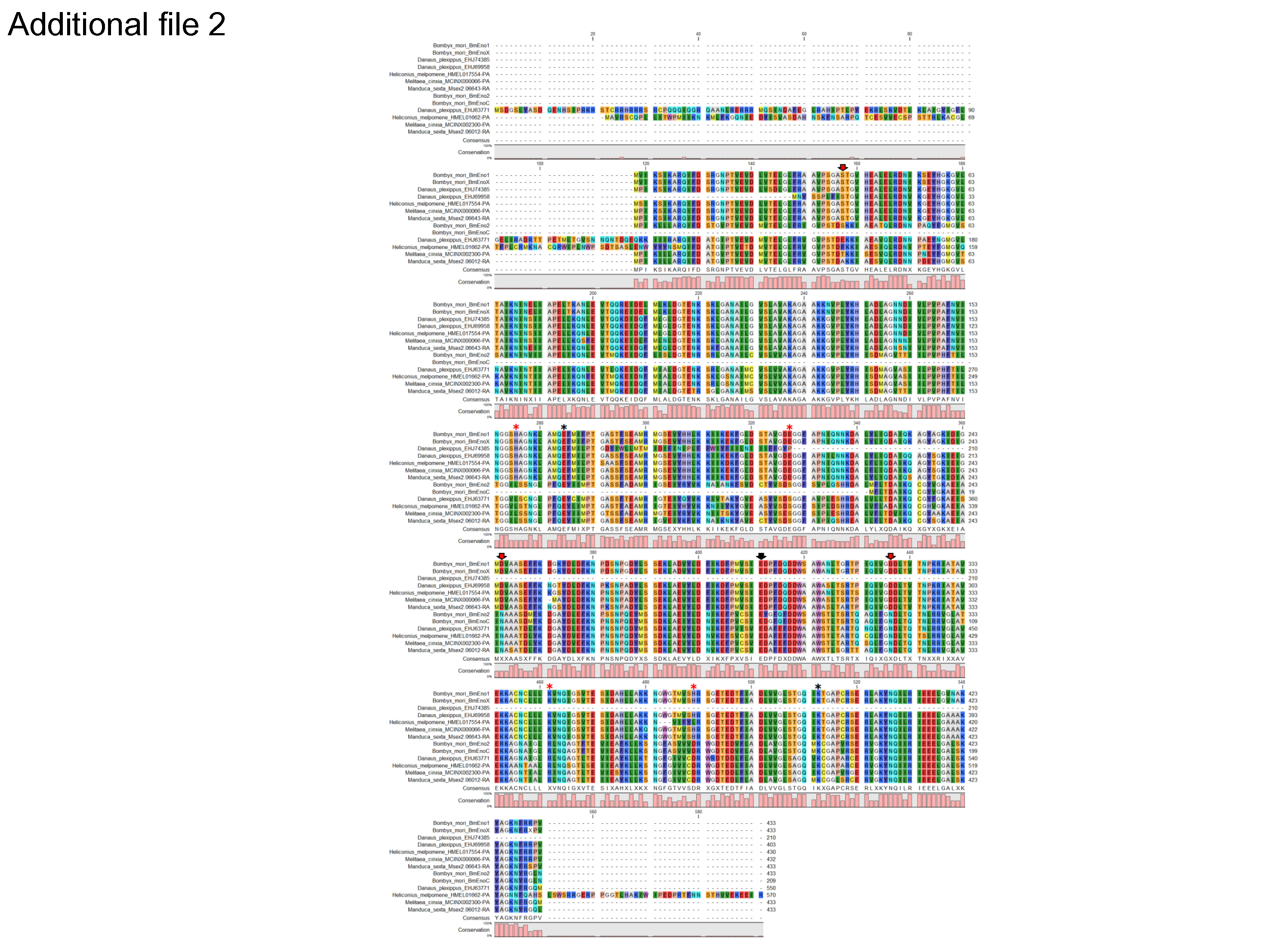

Supplement: Additional file 2: — Amino acid sequence alignment of Lepidopteran enolases. Active site residues are marked with asterisks, and metal-binding residues are labeled with arrows. Red asterisks and arrows indicate amino acid residues that differ among B. mori enolases (BmEnos). The concervation of amino acid residues among the various enolase sequences is graphically shown below the sequence. The residues in the alignment are colored according to the Rasmol color scheme (http://life.nthu.edu.tw/~fmhsu/rasframe/COLORS.HTM#aminocolors). [file 12864_2016_3455_MOESM2_ESM.docx]
